# Clinical, psychological, and sensory characteristics associated with headache attributed to temporomandibular disorder in people with chronic myogenous temporomandibular disorder and primary headaches

**DOI:** 10.1186/s10194-021-01255-1

**Published:** 2021-05-22

**Authors:** Inna E. Tchivileva, Richard Ohrbach, Roger B. Fillingim, Feng-Chang Lin, Pei Feng Lim, Samuel J. Arbes, Gary D. Slade

**Affiliations:** 1 Center for Pain Research and Innovation, Adams School of Dentistry, University of North Carolina at Chapel Hill, Chapel Hill, NC USA; 2 Division of Oral and Craniofacial Sciences, Adams School of Dentistry, University of North Carolina at Chapel Hill, Chapel Hill, NC USA; 3 Department of Oral Diagnostic Sciences, School of Dental Medicine, University at Buffalo, State University of New York, Buffalo, NY USA; 4 Department of Community Dentistry & Behavioral Science, College of Dentistry, University of Florida, Gainesville, FL USA; 5 Department of Biostatistics, Gillings School of Global Public Health, University of North Carolina at Chapel Hill, Chapel Hill, NC USA; 6 Division of Diagnostic Sciences, Adams School of Dentistry, University of North Carolina at Chapel Hill, Chapel Hill, NC USA; 7 Rho Inc, Durham, NC USA; 8 Division of Pediatric and Public Health, Adams School of Dentistry, University of North Carolina at Chapel Hill, Chapel Hill, NC USA

**Keywords:** Orofacial pain, Headache, Migraine, Tension-type headache

## Abstract

**Background:**

Headache attributed to Temporomandibular Disorder (HATMD) is a secondary headache that may have features resulting in diagnostic overlap with primary headaches, namely, tension-type (TTH) or migraine. This cross-sectional study of people with both chronic myogenous TMD and primary headaches evaluated characteristics associated with HATMD.

**Methods:**

From a clinical trial of adults, baseline data were used from a subset with diagnoses of both TMD myalgia according to the Diagnostic Criteria for TMD (DC/TMD) and TTH or migraine according to the International Classification of Headache Disorders, 3rd edition. HATMD was classified based on the DC/TMD. Questionnaires and examinations evaluated 42 characteristics of facial pain, headache, general health, psychological distress, and experimental pain sensitivity. Univariate regression models quantified the associations of each characteristic with HATMD (present versus absent), headache type (TTH versus migraine), and their interaction in a factorial design. Multivariable lasso regression identified the most important predictors of HATMD.

**Results:**

Of 185 participants, 114 (61.6%) had HATMD, while the numbers with TTH (*n* = 98, 53.0%) and migraine (*n* = 87, 47.0%) were similar. HATMD was more likely among migraineurs (61/87 = 70.1%) than participants with TTH (53/98 = 54.1%; odds ratio = 2.0; 95%CL = 1.1, 3.7). In univariate analyses, characteristics associated with HATMD included pain-free jaw opening and examination-evoked pain in masticatory muscles and temporomandibular joints (TMJ) as well as frequency and impact of headache, but not frequency or impact of facial pain. Lowered blood pressure but not psychological or sensory characteristics was associated with HATMD. Multiple characteristics of facial pain, headache, general health, and psychological distress differed between TTH or migraine groups. Few interactions were observed, demonstrating that most characteristics’ associations with HATMD were consistent in TTH and migraine groups. The lasso model identified headache frequency and examination-evoked muscle pain as the most important predictors of HATMD.

**Conclusions:**

HATMD is highly prevalent among patients with chronic myogenous TMD and headaches and often presents as migraine. In contrast to primary headaches, HATMD is associated with higher headache frequency and examination-evoked masticatory muscle pain, but with surprisingly few measures of facial pain, general health, and psychological distress. A better understanding of HATMD is necessary for developing targeted strategies for its management.

**Trial identification and registration:**

SOPPRANO; NCT02437383. Registered May 7, 2015.

**Supplementary Information:**

The online version contains supplementary material available at 10.1186/s10194-021-01255-1.

## Background

Temporomandibular disorders (TMDs) are comorbid with primary headaches. Cross-sectional studies report that the majority of people with TMD have primary headaches [1–5]. Interestingly, the prevalence of headache increased as the number of TMD symptoms increased. The headache prevalence was 56.5% in people with one TMD symptom, 65.1% in people with two TMD symptoms, and 72.8% in people with three or more TMD symptoms [2]. In some studies, migraine was the most prevalent type of headache among TMD patients [6], and migraine prevalence was higher in myogenous compared to arthrogenous TMD [7, 8]. Likewise, many people with headache suffer from TMD. For example, a US population study demonstrated a five-fold higher prevalence of TMD symptoms in people with severe headache compared to those without severe headache (15.6% versus 2.6%) [9]. In a Danish tertiary headache center, the prevalence of TMD in headache patients was 56.1% [10]. Notably, TMD symptoms were greater with an increased frequency of temple headache [4], and the presence of TMD was associated with a greater frequency of migraine episodes [8]. In our prospective cohort of TMD-free individuals, migraine and higher headache frequency at baseline were major risk factors for the subsequent development of TMD [11].

In 2014, the Diagnostic Criteria for TMD (DC/TMD) [12] defined a category of secondary “Headache attributed to TMD” (HATMD). The published diagnostic algorithm for HATMD [13] was also incorporated into the International Classification of Headache Disorders, 3rd edition (ICHD-3) [14]. In the DC/TMD, HATMD is defined as “a headache in the temple area secondary to pain-related TMD that is affected by jaw movement, function, or parafunction, and replication of this headache occurs with provocation testing of the masticatory system” [12]. The diagnostic criteria include the following: history of headache of any type in the temple area; history of the headache modified with jaw movement, function, or parafunction; confirmation by the examiner that the location of headache includes the area defined by the temporalis muscle; and a report of familiar headache in the temple area must be elicited with provocation tests. Unlike the DC/TMD, the ICHD-3 criteria for HATMD did not necessitate the location of headache to the temple area. Furthermore, the ICHD-3 classification included an assessment of the temporal (i.e., chronological) relationship, namely, that the headache developed after the onset of TMD or led to its discovery [14]. In this manuscript, we adhered to the DC/TMD criteria for HATMD.

HATMD occurs frequently in TMD patients, with prevalence ranging from 5.4% to 29.3% in tertiary orofacial pain clinics [15, 16]. However, little is known about the biopsychosocial characteristics that might be associated with HATMD. In our retrospective cohort of patients with painful TMD, patients with HATMD reported a greater number of comorbid pain conditions, exhibited a greater number of painful sites on palpation of the head and neck region, and had higher facial pain intensity compared with patients without HATMD [15]. In patients with myogenous TMD, facial pain intensity between those with and without HATMD was not different, but patients with HATMD had greater pressure pain sensitivity at the anterior temporalis muscle than patients without HATMD [17]. The presence of HATMD may alter the success of TMD management and require distinct treatment interventions. Therefore, a better understanding of this secondary headache is vital.

The aim of this study was to identify clinical, psychological, and sensory characteristics distinguishing between presence versus absence of HATMD in people who had chronic myogenous TMD and primary headaches. A second aim was to determine if the distinguishing characteristics differed for participants with migraine compared with those with TTH. We hypothesized that HATMD was associated with more severe and disabling facial pain, more frequent and impactful headache, higher psychological distress, and greater experimental pain sensitivity. We also assumed that many of the hypothesized associations were augmented in migraineurs compared to participants with TTH.

## Methods

The study’s methods have been described in detail in a separate manuscript [18] and are summarized below.

### Study design

This cross-sectional study was conducted as a part of a multisite, double-blind, placebo-controlled, parallel-group, phase 2b trial (Study of Orofacial Pain and Propranolol, a.k.a. SOPPRANO) that investigated analgesic efficacy of propranolol in patients with chronic myogenous TMD [18]. The trial was approved by the Institutional Review Boards at three sites: the University of North Carolina at Chapel Hill, the University of Florida, and the University at Buffalo. Inclusion criteria aimed to identify people with at least 10 days per month of moderate-to-severe TMD pain lasting at least 3 months, while exclusion criteria were based on contraindications to propranolol therapy and health conditions that may have biased the participants’ pain rating. A complete list of inclusion and exclusion criteria is presented in Additional file 1. All participants provided informed consent at enrollment. The recruitment occurred between August 2015 to January 2018, and the follow-up of the last participant was completed in April 2018. The SOPPRANO trial included a telephone pre-screening which was conducted up to 28 days before the screening visit, a baseline period (lasting 1 to 3 weeks) during which participants were assessed for eligibility, a treatment period (10 weeks), and a follow-up period (1 week).

### Participants

This analysis used baseline data from 185 SOPPRANO participants aged 18 to 65 years who had both chronic myogenous TMD (with or without arthralgia) classified according to the DC/TMD [12] and headache classified according to the ICHD-3 [14].

### Clinical and biopsychosocial characteristics

Clinical indicators of TMD pain were mean weekly facial pain intensity during the day (reported on a 0–100 numeric rating scale) and mean weekly pain duration (reported as the percentage of the waking day with pain). Both facial pain intensity and duration were recorded by the participants in a daily symptom diary. The daily recordings were used to compute the mean weekly scores for the last 7 days of the baseline period. Additional facial pain outcomes obtained from the DC/TMD clinical examination [12] included the classification of myalgia and/or arthralgia, the measurements of pain-free, maximum unassisted, and maximum assisted mouth opening, and the familiar pain responses evoked by examination of the masticatory muscles and temporomandibular joints (TMJ). An examiner calibration session conducted prior to the enrollment demonstrated excellent inter-examiner agreement in the TMD classification (kappa for pairwise comparisons ranged from 0.82 to 1.00). TMD-related disability and interference in functioning were assessed using the Graded Chronic Pain Scale (GCPS) [19] and jaw function was evaluated with the Jaw Functional Limitation Scale (JFLS) [20, 21].

Type of primary headache (migraine or TTH) was assessed via a structured headache interview [11] based on the ICHD-3 [14]. HATMD was determined according to the DC/TMD criteria [12, 13]. The structured headache interview recorded the details of up to 3 different headaches. Information elicited for each headache included the headache location, intensity, quality, duration, frequency, and aggravating factors. The final question asked about an average number of days per month with headache of any type during the past 3 months. For analyses, definite and probable diagnoses of primary headaches were combined into one category for each primary headache type. Participants with both migraine and TTH diagnoses were analyzed as migraine cases. Participants with both HATMD and primary headaches were counted as HATMD cases for the purpose of this study. The Headache Impact Test-6 (HIT-6) was used to assess headache-related disability [22].

General health was assessed with the Short Form-12 Health Survey version 2 (SF-12 v2) [23], which produced composite scores for physical and mental health. Sleep was evaluated with the Pittsburgh Sleep Quality Index (PSQI) [24]. The blood pressure and heart rate were measured with the Accutorr Plus (Datascope Corp., NJ), and were computed as a mean of 3 measurements taken at 2-min intervals following rest in a seated position for 10 min. Psychological characteristics were assessed with the Coping Strategies Questionnaire Revised (CSQ-R) [25], which measured the frequency of engagement in specific coping activities when experiencing pain; the Hospital Anxiety and Depression Scale (HADS) [26], which evaluated anxiety and depression; the Perceived Stress Scale (PSS) [27], which appraised 14 aspects of stress to produce an overall perceived stress rating; and the Symptom Checklist 90-Revised (SCL-90R) [28] somatization subscale, which assessed the somatic symptoms experience.

Pain sensitivity was assessed with heat and pressure tests. Heat pain threshold and tolerance were measured on the ventral forearm using thermal stimulators (Pathway or TSA-II; Medoc; Ramat Yishai, Israel) accessorized with a 16 × 16 mm thermode. The cutoff temperature for both measurements was 50 °C. Following a pre-trial test, the average values were calculated from 4 tests conducted with a 5-s inter-stimulus interval at different sites on the ventral forearm. Pressure pain thresholds (PPTs) were assessed bilaterally over the temporalis, masseter, and trapezius muscles, the TMJ, and the lateral epicondyles with a pressure algometer (FDX-10, Wagner Instruments, CT). One pre-trial assessment was performed at each site, followed by additional assessments until 2 measures differing by less than 0.2 kg were obtained or 5 assessments were administered. The mean of the 2 closest values was determined, and the values from the right and left sides were averaged to obtain a single PPT per anatomical site. In the OPPERA study which used the same methodology for PPT assessment, PPTs measured at both cranial and non-cranial sites were the most robust sensory testing modality for prediction of chronic TMD, and examiners exhibited excellent reliability in quantifying the thresholds [29].

### Statistical methods

Two binary variables – HATMD (presence or absence) and headache type (TTH or migraine) – defined four groups of participants in a 2 × 2 matrix and were used as independent variables along with their interactions in univariate regression models. For descriptive purposes, 37 continuous variables were summarized as means and standard deviations within each group, while percentages were calculated for 5 binary characteristics (namely, gender, race, ethnicity, TMD-related disability, and chronic headache). Each characteristic was then used as the dependent variable in univariate regression models (linear regression for the continuous characteristics and binary logistic regression for the binary characteristics). *P* values for the univariate associations were reported for all variables for descriptive purposes only. The Bonferroni-adjusted threshold for correction for multiple comparisons would be *P* ≤ 0.001 (i.e., 0.05/42). While Bonferroni correction provides sufficient protection against type I error for traditional judgments about statistical significance in the presence of multiple tests, *P* values from univariate associations are an invalid criterion for the selection of variables for multivariable modeling [30]. Instead, we adopted a more advanced approach of lasso regression for multivariable modeling. Specifically, two models within a lasso regression framework [31, 32] were used to identify the most important predictors of HATMD. Lasso regression was used because it is superior to regular logistic regression which tends to over-fit the data in the presence of a large number of predictor variables, particularly when they are highly correlated, as occurs in this study.

The first lasso regression model aimed to identify a small number of variables that predict HATMD well. Using the “lasso logit” command in Stata [32], a binary logistic regression lasso model with cross-validation was created using all 42 characteristics as predictor variables. To aid in the interpretation, all continuous variables were transformed to z-scores with mean of 0 and standard deviation of 1. The model’s penalized coefficients with non-zero values were reported, with larger absolute values indicating the more important predictor variables. In contrast, the remaining variables all have coefficients of zero, signifying that they make no meaningful contribution to predicting HATMD beyond the prediction provided by variables that have non-zero coefficients.

The second lasso regression model estimated multivariable-adjusted odds ratios for two of the variables with non-zero coefficients. This was achieved with the “lasso dslogit” command in Stata that uses double-selection models [32] for inference. The two variables were selected because they were hallmarks of TMD (i.e., familiar examination-evoked masticatory muscle pain) and of headache (i.e., the number of days with headache in the preceding month). The lasso model used the other 40 characteristics as control variables, hence creating adjusted odds ratios for familiar-evoked pain and the number of days with headache. All continuous variables were again transformed to z-scores. This model generated parameter estimates and standard errors for the two main predictor variables from a double-selection process, producing estimates that can be interpreted in the same way as estimates from a conventional logistic regression model. Odds ratios (OR) and 95% confidence limits (CL) were therefore reported for the two main predictor variables.

## Results

### Participants

**Fig. 1:**
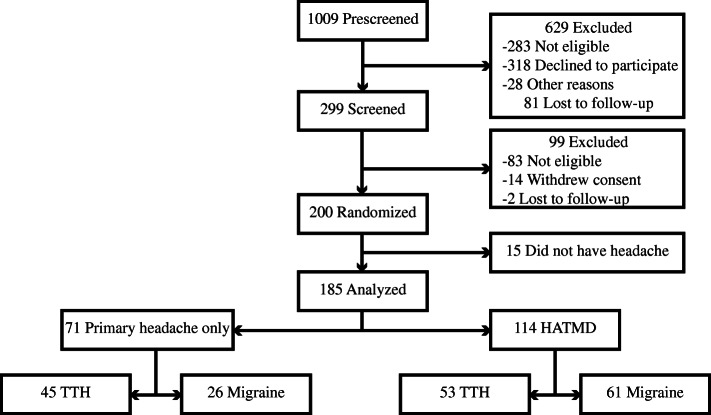
Flowchart. Abbreviations: HATMD, headache attributed to TMD; TTH, tension-type headache

Of the 200 participants enrolled in the SOPPRANO trial for treatment of chronic myogenous TMD, 185 (92.5%) met ICHD-3 criteria [14] for TTH and/or migraine and were included in the current study (Fig. 1). In 114 (61.6%) participants, their primary headaches also satisfied DC/TMD criteria [12] for HATMD. Most of the participants were young white females, and their demographic characteristics did not differ according to the presence or absence of HATMD or type of primary headache (Table 1).

**Table 1:** Demographic and clinical characteristics in four groups classified according to primary headache and HATMD^a^

Characteristic	Type of primary HA				***P*** value^**c**^		
	TTH		Migraine				
	Primary HA only ***n*** = 45	HATMD ***n*** = 53	Primary HA only ***n*** = 26	HATMD ***n*** = 61	Effect of HATMD	Effect of HA type	Interaction
DEMOGRAPHIC							
Age, years	35.4 (14.9)	32.3 (12.1)	36.1 (14.4)	33.7 (10.6)	0.167	0.572	0.856
Sex, female, n (%)	32 (71.1)	42 (79.3)	22 (84.6)	49 (80.3)	0.858	0.269	0.350
Race, white, n (%)	29 (64.4)	46 (86.8)	20 (76.9)	49 (80.3)	0.051	0.861	0.155
FACIAL PAIN							
Time since onset, years	8.0 (9.5)	10.3 (8.6)	14.4 (14.0)	11.2 (7.7)	0.754	**0.019**	0.074
Frequency in the last 30 days, d	23.5 (7.2)	24.2 (6.9)	23.7 (7.0)	23.1 (6.9)	0.975	0.658	0.519
Myalgia with arthralgia, n (%)	42 (93.3)	50 (94.3)	23 (88.5)	59 (96.7)	0.230	0.980	0.354
Mean weekly pain intensity, 0–100 scale	45.1 (16.2)	45.8 (14.6)	48.8 (14.2)	50.5 (16.0)	0.618	0.081	0.820
Mean weekly pain duration, % of waking day	57.9 (27.1)	58.0 (24.1)	52.8 (22.7)	62.9 (26.8)	0.198	0.975	0.207
GCPS: Characteristic pain intensity, 0–100 scale	57.2 (13.9)	55.6 (13.9)	58.2 (15.5)	64.8 (15.1)	0.266	**0.024**	0.070
GCPS: Facial pain interference, 0–100 scale	22.5 (22.4)	19.2 (19.5)	29.1 (26.6)	39.5 (23.8)	0.316	**<.001**	0.052
GCPS grade IIb-IV, n (%)	13 (33.3)	12 (22.6)	12 (46.2)	41 (67.2)	0.611	**<.001**	**0.033**
Pain-free jaw opening, mm	31.7 (10.7)	28.2 (10.4)	31.3 (12.1)	27.7 (11.5)	**0.040**	0.801	0.984
Maximum unassisted jaw opening, mm	45.6 (8.5)	42.5 (9.6)	45.5 (9.7)	43.3 (9.7)	0.072	0.835	0.773
Maximum assisted jaw opening, mm	49.6 (8.6)	47.1 (8.7)	48.8 (12.1)	47.3 (9.1)	0.170	0.843	0.732
Familiar muscle pain responses on examination, 0-24^b^	8.0 (3.1)	9.2 (3.0)	7.8 (4.4)	10.5 (3.7)	**0.001**	0.326	0.199
Familiar TMJ pain responses on examination, 0–14	5.8 (3.5)	7.1 (3.5)	6.7 (4.4)	8.0 (3.8)	**0.025**	0.119	0.994
JFLS total score, 0–10 scale	2.3 (1.5)	2.4 (1.5)	2.6 (1.4)	3.2 (1.9)	0.145	**0.033**	0.362
HEADACHE							
Time since onset, y	16.3 (11.6)	12.6 (9.6)	18.9 (14.2)	16.3 (8.9)	0.056	0.055	0.753
Monthly frequency in the last 3 months, d	10.0 (9.4)	14.3 (8.8)	13.9 (10.9)	19.2 (8.7)	**0.001**	**0.002**	0.718
Chronic headache, n (%)	10 (22.2)	25 (47.2)	7 (26.9)	22 (36.1)	**0.023**	0.765	0.299
HIT-6 score, 36–78 scale	51.0 (7.9)	53.5 (7.0)	58.8 (7.3)	60.6 (5.8)	**0.048**	**<.001**	0.738
GENERAL HEALTH							
SF-12v2 Physical Health, 0–100 scale	50.7 (9.1)	52.5 (8.8)	46.4 (10.0)	45.7 (11.1)	0.720	**<.001**	0.422
PSQI total score, 0–21 scale	6.4 (3.8)	6.0 (3.7)	6.8 (3.9)	7.6 (3.3)	0.794	0.084	0.287
Heart rate, bpm	70.5 (10.9)	68.7 (11.7)	74.9 (10.7)	71.7 (11.8)	0.163	**0.038**	0.691
Systolic blood pressure, mm Hg	122.4 (12.5)	118.5 (12.1)	128.8 (12.3)	120.6 (14.9)	**0.004**	**0.039**	0.286
Diastolic blood pressure, mm Hg	73.2 (9.2)	70.1 (8.9)	78.5 (10.0)	72.9 (9.6)	**0.003**	**0.006**	0.393

*Abbreviations*: *GCPS* Graded Chronic Pain Scale, *HA* Headache, *HATMD* Headache attributed to TMD, *HIT-6* Headache Impact Test-6, *JFLS* Jaw Functional Limitation Scale, *PSQI* Pittsburgh Sleep Quality Index, *SD* Standard deviation, *SF-12v2* Short-Form 12 Health Survey version 2, *TMD* Temporomandibular disorder, *TMJ* Temporomandibular joint

^a^Data are mean (SD) unless otherwise indicated

^b^Pain responses from examination of temporalis and masseter muscles

^c^*P* values < 0.05 are highlighted in a bold font

### Type of primary headache and HATMD

Ninety-eight (53.0%) participants met criteria for probable or definite TTH, and 87 (47.0%) met criteria for probable or definite migraine (with or without coexisting TTH) (Table 1). The proportion of migraineurs was higher among participants with HATMD than participants with primary headache only (53.5% versus 36.6%, *P* = 0.025; OR = 2.0, 95% CL = 1.1, 3.7).

### Facial pain characteristics associated with HATMD and type of primary headache

Most of the 14 characteristics of facial pain did not differ based on the presence or absence of HATMD (Table 1). The exceptions were pain-free jaw opening, which was reduced in the presence of HATMD (*P* = 0.040), the number of familiar examination-evoked masticatory muscle pain responses (*P* = 0.001), and the number of familiar examination-evoked TMJ pain responses (*P* = 0.025), both of which were elevated in the presence of HATMD. Multiple facial pain characteristics were associated with type of primary headache (Table 1). Compared with TTH participants, migraineurs reported longer time since onset of facial pain (*P* = 0.019), higher characteristic facial pain intensity (*P* = 0.024), and greater facial pain interference (*P* < 0.001), as measured by the GCPS. Moreover, migraine was associated with greater likelihood of disabling TMD, namely, GCPS grades ranging from IIb to IV (*P* < 0.001). Finally, migraineurs had a higher JFLS total score (*P* = 0.033). Interaction between HATMD and type of primary headache was observed for only one facial pain characteristic, the TMD GCPS grade (*P* = 0.033), and the relationship was antagonistic. Among migraineurs, HATMD was associated with greater likelihood of disabling TMD, whereas among TTH participants, HATMD was associated with lower likelihood of disabling TMD.

In summary, the type of primary headache, not the HATMD status, was associated with more severe and debilitating facial pain.

### Headache characteristics associated with HATMD and type of primary headache

Compared with participants with primary headaches in the absence of HATMD, participants with HATMD had more frequent (*P* = 0.001) and chronic (*P* = 0.023) headaches, and headaches had a greater impact on their life (*P* = 0.048) (Table 1).

Migraine was associated with higher total frequency of headache of any type (*P* = 0.002) and more impact (*P* < 0.001) than TTH. The prevalence of chronic headache did not differ between migraineurs and TTH participants (*P* = 0.765). The HATMD status and type of primary headache did not interact in their effect on headache characteristics.

Overall, participants with HATMD or migraine had more frequent and debilitating headaches.

### Characteristics of general health associated with HATMD and type of primary headache

Participants with HATMD did not differ from participants with only primary headache in measures of physical health and quality of sleep but had lower systolic and diastolic blood pressure (*P* = 0.004 and *P* = 0.003, respectively) (Table 1). Compared with TTH participants, migraineurs had poorer physical health (*P* < 0.001), greater heart rate (*P* = 0.038), and higher systolic and diastolic blood pressure (*P* = 0.039 and *P* = 0.006, respectively). No interaction effect on general health characteristics was found for HATMD and the type of primary headache. In summary, both the HATMD status and type of primary headache were associated with cardiovascular characteristics.

### Psychological and sensory characteristics associated with HATMD and type of primary headache

Psychological characteristics and measures of experimental pain sensitivity did not distinguish HATMD participants from participants with primary headache only (Table 2). In contrast, the type of primary headache was strongly associated with psychological factors but not with sensory characteristics. Compared with TTH participants, migraineurs had higher scores on several CSQ-R subscales, including pain catastrophizing (*P* = < 0.001), and they reported greater anxiety (*P* = 0.008), perceived stress (*P* = 0.020), and somatic symptoms (*P* < 0.001).

**Table 2:** Psychological and QST characteristics in four groups classified according to primary headache and HATMD^a^

Characteristic	Type of primary HA				***P*** value^**b**^		
	TTH		Migraine				
	Primary HA only ***n*** = 45	HATMD ***n*** = 53	Primary HA only ***n*** = 26	HATMD ***n*** = 61	Effect of HATMD	Effect of HA type	Interaction
PSYCHOLOGICAL							
CSQ-R catastrophizing, 0–6 scale	0.9 (1.0)	0.8 (0.8)	1.3 (1.2)	1.6 (1.4)	0.547	**<.001**	0.209
CSQ-R distraction, 0–6 scale	2.2 (1.3)	2.1 (1.5)	2.4 (1.7)	2.8 (1.7)	0.611	**0.044**	0.313
CSQ-R praying, 0–6 scale	2.1 (2.2)	1.3 (1.8)	2.4 (2.2)	2.6 (2.0)	0.353	**0.006**	0.117
CSQ-R ignoring pain, 0–6 scale	3.1 (1.6)	3.1 (1.5)	3.4 (1.3)	2.5 (1.5)	0.086	0.639	0.052
CSQ-R distancing, 0–6 scale	1.2 (1.5)	1.0 (1.4)	1.6 (1.7)	1.4 (1.5)	0.405	0.074	0.913
CSQ-R coping self-statements, 0–6 scale	4.2 (1.5)	3.7 (1.4)	4.4 (1.2)	4.3 (1.3)	0.178	0.096	0.353
HADS anxiety, 0–21 scale	6.8 (4.2)	6.1 (3.7)	7.3 (4.5)	9.1 (4.6)	0.457	**0.008**	0.058
HADS depression. 0–21 scale	3.2 (3.5)	2.9 (3.0)	3.0 (2.8)	4.8 (3.6)	0.135	0.094	**0.040**
Perceived stress scale, 0–56 scale	20.5 (8.3)	19.1 (7.8)	20.6 (8.9)	25.5 (10.0)	0.224	**0.020**	**0.026**
SCL-90R somatization, 0–4 scale	0.5 (0.4)	0.5 (0.3)	0.7 (0.6)	0.9 (0.5)	0.130	**<.001**	0.399
SF-12v2 Mental Health, 0–100 scale	48.7 (10.1)	51.3 (9.6)	50.5 (9.1)	44.9 (11.6)	0.356	0.152	**0.013**
QUANTITATIVE SENSORY TESTING							
Heat pain threshold, 32–50 °C	41.7 (3.3)	41.5 (3.9)	42.0 (2.9)	40.8 (3.5)	0.174	0.726	0.376
Heat pain tolerance, 32–50 °C	45.6 (3.4)	45.9 (3.3)	46.0 (1.9)	45.2 (3.3)	0.614	0.725	0.264
Temporalis PPT, 0–500 kPa	107.3 (52.7)	105.0 (62.3)	121.9 (59.4)	99.3 (57.0)	0.186	0.577	0.284
Masseter PPT, 0–500 kPa	101.0 (51.1)	102.7 (59.8)	112.8 (52.4)	90.7 (55.0)	0.249	0.978	0.178
TMJ mean PPT, 0–500 kPa	91.1 (47.3)	95.3 (53.7)	106.7 (54.0)	88.2 (53.7)	0.407	0.568	0.179
Trapezius PPT, 0–500 kPa	216.0 (94.8)	201.2 (116.5)	205.8 (99.0)	181.6 (117.7)	0.271	0.406	0.813
Lateral epicondyle PPT, 0–500 kPa	214.0 (85.8)	214.7 (115.9)	232.9 (108.6)	194.4 (106.2)	0.252	0.973	0.234

*Abbreviations*: *CSQ-R* Coping Strategies Questionnaire-Revised, *HA* Headache, *HADS* Hospital Anxiety and Depression Scale, *HATMD* Headache attributed to TMD, *PPT* Pressure pain threshold, *SCL-90R* Symptom Checklist 90-Revised, *SD* Standard deviation, *SF-12v2* Short-Form 12 Health Survey version 2, *TMD* Temporomandibular disorder, *TMJ* Temporomandibular joint, *QST* Quantitative sensory testing

^a^Data are mean (SD)

^b^*P* values < 0.05 are highlighted in a bold font

A statistically significant interaction between HATMD and type of primary headache was observed for 3 psychological characteristics: depression (*P* = 0.040), perceived stress (*P* = 0.026), and overall mental health (*P* = 0.013). HATMD was associated with greater depression, higher stress, and poorer mental health in migraineurs, but in TTH participants, HATMD was associated with lower depression, reduced stress, and better mental health.

Overall, HATMD was not associated with psychological characteristics, while migraine was. However, migraineurs whose migraine satisfied criteria for HATMD had poorer psychological characteristics than migraineurs without HATMD. Sensory characteristics were not contingent on HATMD status or type of primary headache.

### Predictors of HATMD from a multivariable statistical model

Out of 42 demographic, clinical, psychological, and sensory characteristics, the lasso regression model identified 12 variables with non-zero penalized coefficients, namely, three facial pain characteristics, three headache characteristics, two measures of blood pressure, and two psychological characteristics (Table 3). The total frequency of headache and the number of familiar examination-evoked masticatory muscle pain responses had the largest absolute values, signifying greater importance in predicting HATMD. The ignoring-pain subscale of CSQ-R and the pain-free jaw opening, on the contrary, had the smallest coefficients, signifying lesser importance. In the inferential model that adjusted for all other potential covariates, the number of familiar examination-evoked masticatory muscle pain responses was associated with greater odds of HATMD (OR = 2.98; 95% CL = 1.58, 5.62, associated with an increase of three standard deviations in the number of responses), as was the frequency of headache (OR = 1.49; 95% CL = 1.02, 2.17, associated with an increase of two standard deviations in headache frequency).

**Table 3:** Multivariable lasso regression model of characteristics distinguishing between presence versus absence of HATMD

Predictor	Penalized coefficient^a^	Inferential OR (95% CL)^b^
Monthly headache frequency in the last 3 months	0.311	1.49 (1.02, 2.17)
Familiar masticatory muscle pain responses on examination	0.242	2.98 (1.58, 5.62)
Systolic blood pressure	−0.141	n/a
HIT-6 score	0.130	n/a
CSQ-R coping self-statements	−0.119	n/a
Diastolic blood pressure	−0.097	n/a
Time since headache onset	−0.067	n/a
Familiar TMJ pain responses on examination	0.063	n/a
Maximum unassisted jaw opening	−0.059	n/a
Migraine (headache type)	0.057	n/a
Pain-free jaw opening	−0.012	n/a
CSQ-R ignoring pain	−0.004	n/a

*Abbreviations*: *CSQ-R* Coping Strategies Questionnaire-Revised, *HIT-6* Headache Impact Test-6, *TMD* Temporomandibular disorder, *TMJ* Temporomandibular joint

^a^ Penalized coefficients are non-zero values from the Stata “lasso logit” command that used 42 potential predictors variables. (Variables not listed have coefficients of zero.)

^b^ Odds ratios are from the Stata “dslogit” command that specified two a priori predictor variables (monthly headache frequency in the last 3 months and familiar muscle pain responses on examination) with the remaining 40 potential predictors as control variables. The command uses a double-selection process for inference yielding odds ratios (OR) and 95% confidence limits (CLs) for the two a priori variables

## Discussion

### Summary of the main findings

In this study of adults with chronic myogenous TMD and primary headache, 61.6% of participants met DC/TMD criteria for HATMD. The odds of HATMD were elevated two-fold in migraineurs compared to people with TTH. In the univariate analyses, HATMD was associated with greater frequency and impact of headache, but not greater frequency or impact of facial pain. Contrary to our hypothesis, no association was found with psychological or sensory characteristics. Of note, multiple characteristics of facial pain, headache, general health, and psychological distress were associated with the type of primary headache. Few interactions were observed, demonstrating that most characteristics’ associations with HATMD were consistent in the TTH and migraine groups. Multivariable analysis identified headache frequency and familiar examination-evoked masticatory muscle pain as the most important predictors of HATMD.

### Comparison with previously published studies and potential biological mechanisms

Despite the development of validated criteria for the diagnosis of HATMD, the distinction of myogenous TMD, primary headache, and HATMD remains elusive and challenging. The extensive overlap between painful TMD and primary headaches had been reported in many cross-sectional studies [1–4, 33] and can be explained by the shared trigeminal pain pathway with neuroanatomical connectivity between the three branches of the trigeminal nerve as well as by peripheral and central sensitization [34]. This shared pathophysiology densifies the complex comorbidity of TMD and headaches, especially with respect to the etiology. Our seven-year prospective cohort study of TMD-free people identified migraine and higher headache frequency as the major risk factors for the first onset of TMD [11]. In parallel, a two-year prospective study found that the presence of TMD at baseline predicted the future onset of headaches [35]. This data indicate the bi-directional phenomenon in the continuum of chronic head and face pain.

TMD as a cause of headache was first recognized in the ICHD-2. The diagnostic criteria for HATMD underwent revision by the Validation Project [13] and were reflected in the DC/TMD [12] and ICHD-3 [14]. The present study further characterized HATMD via identification of characteristics which distinguished HATMD from primary headaches. One significant finding was that HATMD often presented as migraine. Previously, large population-based studies have demonstrated greater association of TMD with migraine than episodic TTH [2], and the presence of TMD was associated with increased migraine frequency and greater use of migraine medication [8].

Since TMD is a necessary cause of HATMD, it is reasonable to assume that HATMD could be a consequence of more severe and frequent facial pain. Contrary to this hypothesis, HATMD was not associated with greater intensity, frequency, or impact of facial pain in this cohort. In the univariate analyses of many measured characteristics of facial pain, HATMD was associated only with pain-free jaw opening and familiar examination-evoked pain of masticatory muscles and TMJ. While the association with evoked pain of masticatory muscles and TMJ is consistent with our previous report [15], the lack of association with facial pain intensity is not. The discrepancy could be due to differences in criteria used to select study participants. In our earlier study, the control group included TMD patients without any headache that could enhance the difference in facial pain intensity between cases with HATMD and controls without HATMD. Like in this study, no difference in baseline facial pain intensity was found between patients with and without HATMD in another study of myogenous TMD [17].

HATMD was positively associated with multiple characteristics of headache, namely, greater headache frequency, a higher HIT-6 score, and headache chronicity. Given the causal relation between TMD and HATMD, the implication is that HATMD should be responsive to TMD treatment. In a clinical trial comparing efficacy of behavioral therapy with and without occlusal appliance therapy in patients with both myogenous TMD and HATMD, headache intensity and frequency were reduced in both groups with no statistically significant differences between the groups [36]. This implied that HATMD was responsive to the behavioral management of TMD, but the addition of an oral appliance did not offer any further benefit. However, several clinical trials have suggested the benefit of occlusal appliance therapy in the treatment of headache in the presence of TMD [37–39], but these trials have not specifically investigated HATMD.

As expected, multiple characteristics of facial pain, headache, general health, and psychological distress were augmented in migraine compared with TTH. Migraine is more debilitating than TTH and has a higher magnitude of comorbidity with anxiety and depression than TTH [40]. A lack of an association of experimental pain sensitivity with the type of primary headache is also consistent with previous reports. Both migraine and TTH are accompanied by craniofacial muscle tenderness, and the craniofacial pressure pain thresholds do not differ between these headaches [41].

### Strengths and limitations

An important strength of this study was the use of the validated DC/TMD [12] for the classification of TMD myalgia and arthralgia, and the use of the structured, ICHD-3-based interview for classification of headache [11]. All participants had chronic myogenous TMD with moderate to severe facial pain, which made the study sample generalizable to clinic patient population. This study intentionally evaluated a large number of characteristics for their possible association with HATMD with the recognition that TMD and headache are influenced by a multitude of factors. To integrate this vast array of multiple interacting factors, we employed the lasso regression. The lasso model identified total frequency of headache and familiar examination-evoked masticatory muscle pain as the most important predictors of HATMD. While familiar examination-evoked pain in temporalis muscle is already included in the diagnostic criteria for HATMD, headache frequency need to be validated in future studies for possible inclusion in the diagnostic criteria.

There are several limitations in this study. Although the headache diagnostic criteria were determined through a structured interview, the use of daily headache diaries for classification is superior. However, we were not able to incorporate questions relevant for headache diagnosis into our daily symptom diaries due to concern regarding participant burden. Furthermore, we relied on retrospective assessment of headache frequency, because utilizing a more precise, diary-based prospective assessment would have required a longer baseline period and could have led to a greater participant dropout. Additionally, the cross-sectional study design did not permit interpretation of the temporal relationship between HATMD and the putative biopsychosocial causes.

## Conclusions

HATMD is highly comorbid with chronic myogenous TMD and often presents as migraine. In contrast to primary headaches, HATMD is characterized by higher headache frequency and familiar examination-evoked pain of the masticatory muscles. Surprisingly, HATMD was not associated with facial pain, general health, psychological distress, and experimental pain sensitivity. A better understanding of HATMD is necessary for developing targeted strategies for its management.

## Supplementary Information

**Additional file 1.**

## Data Availability

The datasets used in the current study are available from the corresponding author on reasonable request.

## Abbreviations

CL: Confidence limits
CSQ-R: Coping Strategies Questionnaire-Revised
DC/TMD: The Diagnostic Criteria for TMD
GCPS: Graded Chronic Pain Scale
HA: Headache
HADS: Hospital Anxiety and Depression Scale
HATMD: Headache attributed to TMD
HIT-6: Headache Impact Test-6
ICHD: The International Classification of Headache Disorders
JFLS: Jaw Functional Limitation Scale
OR: Odds ratio
PPT: Pressure pain threshold
PSQI: Pittsburgh Sleep Quality Index
SCL-90R: Symptom Checklist 90-Revised
SD: Standard deviation
SF-12v2: Short-Form 12 Health Survey version 2
SOPPRANO: Study of Orofacial Pain and Propranolol
TMD: Temporomandibular disorder
TMJ: Temporomandibular joint
TTH: Tension-type headache
QST: Quantitative sensory testing
