# Supplementary material for: Clinical, psychological, and sensory characteristics associated with headache attributed to temporomandibular disorder in people with chronic myogenous temporomandibular disorder and primary headaches
Source: J Headache Pain. 2021 May 22;22(1):42. doi: 10.1186/s10194-021-01255-1 (PMC8141151; doi:10.1186/s10194-021-01255-1)
Supplement: Supplementary file 1 — Additional file 1. [file 10194_2021_1255_MOESM1_ESM.docx]

## List of Inclusion and Exclusion Criteria for SOPPRANO Trial

## Participant Inclusion Criteria

A participant must meet all of the following inclusion criteria to be eligible for initial enrollment and randomization:

1. Provides a signed and dated informed consent form
2. Is between 18 and 65 years of age (inclusive; male or female and any race or ethnicity)
3. Meets diagnostic criteria for TMD, Group II: Masticatory Muscle Disorders, 1A: Myalgia
4. Has experienced facial pain for at least 3 months
5. Has experienced facial pain for at least 10 days of the last 30 days
6. At Screening and Baseline Visit (Visit 0), reports an average pain intensity rating over the past week of ≥ 30 on a numerical rating scale (0-100)
7. At randomization (Visit 1), has satisfactorily completed at least 4 entries per week in the Daily Symptom Diary within the preceding week and reported an average pain intensity score ≥ 30 on a numerical rating scale (0-100) or reports a rating of average daily pain intensity of ≥30 on the same scale on at least 3 days over the past week
8. If taking a prescription medication (with the exception of prescription formulations of NSAIDs, acetaminophen, and aspirin) episodically (or as needed) for the management of pain, agrees to discontinue its use prior to or at the Screening and Baseline Visit
9. If taking a prescription medication daily for the management of pain, agrees to continue the daily use of the medication throughout the study
10. If taking an over-the-counter pain medication daily, agrees to continue the daily use of the medication throughout the study
11. Agrees to not commence any new prescription medication for the management of pain throughout the study
12. Agrees to not commence any injection therapy for pain (e.g., tender or trigger point injections, steroid injections) during the course of the study
13. Agrees to not use acupuncture, biofeedback, or transcutaneous electrical nerve stimulation (TENS) for the management of pain during the course of the study
14. Agrees to not commence occlusal splint therapy during the course of the study
15. Agrees to continue use of splint therapy if commenced more than 30 days previously
16. Females agree to limit consumption of alcohol to no more than 7 drinks and males agree to limit consumption of alcohol to no more than 14 drinks a week for the duration of the study
17. Females of childbearing potential, agrees to use one of the following methods of contraception throughout the study: licensed hormonal method, intrauterine device, condoms with contraceptive foam, abstinence, or vasectomy in partner (if post‑menopausal, must not have menstruated for at least 12 consecutive months)
18. Willing and able to understand and comply with all study procedures and be available for the duration of the study

## Participant Exclusion Criteria

A potential participant who meets any of the following criteria will be excluded from enrollment and randomization:

1. Has a history of congestive heart failure or the presence of any of the following cardiovascular conditions: clinically significant abnormal 12-lead ECG, sinus bradycardia (resting heart rate below 55 beats per minute although lower heart rates will be acceptable upon the review and approval by the site cardiologist), greater than first degree heart block, coronary artery disease, uncontrolled hypertension, or hypotension (systolic blood pressure below 90 mm Hg). In individuals with a heart rate below 55 beats per minute, the site cardiologist will review the participant’s medical history, including any concomitant medications, to ensure that they are in good general health and have no medical contraindications to taking propranolol.
2. Has any of the following medical conditions: bronchial asthma, nonallergic bronchospasm (chronic obstructive pulmonary disease and emphysema), renal failure or dialysis, diabetes mellitus, hyperthyroidism, fibromyalgia, or uncontrolled seizures
3. Has known hypersensitivity to propranolol or components of the placebo capsules
4. Currently taking a β-blocker or medication that may interact with propranolol, including haloperidol, intravenous verapamil, and catecholamine-depleting drugs, such as reserpine, within 30 days prior to the Screening and Baseline Visit
5. Currently taking an opioid medication, whether episodically or daily, within 30 days prior to the Screening and Baseline Visit
6. Has commenced a new daily prescription medication for the management of pain within 30 days prior to the Screening and Baseline Visit
7. Used any injection therapy (e.g., tender or trigger point injections, steroid injections) for the management of pain within 2 weeks prior to the Screening and Baseline Visit
8. Used acupuncture, biofeedback, or TENS for the management of pain within 2 weeks prior to the Screening and Baseline Visit
9. Has commenced occlusal splint therapy for the management of facial pain within 30 days prior to the Screening and Baseline Visit
10. Has experienced facial trauma or orofacial surgery within 6 weeks prior to the Screening and Baseline Visit
11. Is undergoing current active orthodontic treatment (passive retainers are permitted)
12. Has a history of major depression or other major psychiatric disorder requiring hospitalization within the last 6 months prior to the Screening and Baseline Visit
13. Has a history of treatment for drug or alcohol abuse within the last year
14. Has scored 8 or more on the Alcohol Use Disorders Identification Test (AUDIT) at the Screening and Baseline Visit
15. Currently smokes at least 25 cigarettes per day
16. Currently being treated with chemotherapy or radiation therapy
17. Has been treated with another investigational drug or treatment within 30 days prior to the Screening and Baseline Visit
18. Is pregnant or nursing
19. Anything that, in the opinion of the investigator, would place the participant at increased risk or preclude the participant’s full compliance with or completion of the study
